# Torsional Behavior of Waste Fiber-Reinforced Concrete

**DOI:** 10.3390/ma17133269

**Published:** 2024-07-02

**Authors:** Artur Sanok, Jacek Domski, Janusz Kobaka, Dominik Logoń

**Affiliations:** 1 Faculty of Civil Engineering, Environmental and Geodetic Sciences, Koszalin University of Technology, 75-453 Koszalin, Poland; artur.sanok@tu.koszalin.pl; 2 Faculty of Geoengineering, University of Warmia and Mazury in Olsztyn, 10-720 Olsztyn, Poland; 3 Faculty of Civil Engineering, Wroclaw University of Science and Technology, 50-370 Wroclaw, Poland; dominik.logon@pwr.edu.pl

**Keywords:** concrete, torsional properties, steel fiber

## Abstract

Factory made steel fiber and steel fiber derived from worn tires was used to develop cement concrete, which was subjected to torsional forces. A dedicated stand for torsion tests, allowing for the measurement of force, deflection, and torsion angle, was used. The test results showed that both the factory-made fiber and the waste steel fiber significantly improved torsional properties of the concrete matrix. The test results of specimens made with waste fiber were characterized by slightly worse results compared to factory-made fibers, but there was a significant improvement in torsional properties compared to samples without fibers. Taking into account the financial and environmental benefits, the application of waste steel fiber recovered from car tires could be an interesting alternative to using commercially sold steel fiber applied for the production of construction elements subjected to torsional forces.

## 1. Introduction

In recent times, much attention has been paid to environmental protection. Energy saving, reduction in CO_2_ emission, and reuse of materials contained in waste products are becoming topics that are gaining popularity. The materials that have completed their service life and are disposed of in landfills have a value that is yet to be determined. The reuse of these materials has become a priority of the modern economy in sustainable development times. The construction industry has great potential in the field of management and utilization of waste, especially regarding concrete as a material used worldwide with significant capability of utilizing other waste materials [1,2,3,4]. Waste materials can also be obtained from other sources than the construction industry: silica fume, a material added to concrete since the beginning of the 50s of the 20th century, is a by-product in the production of silicon and ferrosilicon alloys, fly ash, used as a partial substitute material for Portland cement, is a coal combustion product. Another example is the automotive industry, which has achieved unprecedented development in the last few decades. Worldwide, the number of cars has increased significantly. This involves the management of used car parts and the need to reuse materials. Car tires are a good example of reusable vehicle components. Modern tires are reinforced with steel fibers that can be successfully recovered. Waste tire steel fiber (WTSF) after cutting can be successfully used in the production of steel fiber-reinforced concrete [5,6,7]. In the research work conducted for the purpose of this paper, steel fibers recovered from car tires were used.

Because concrete is a brittle material [8,9,10,11], the process of its destruction is rapid and potentially dangerous for people in the case of a construction disaster. One of the means to improve this property is to reduce brittleness by adding fibers to the concrete matrix. Fiber-reinforced concrete (FRC), after the hardening process, is characterized by significantly higher tensile strength, higher dynamic modulus of elasticity, improves post-cracking properties under tension [12], and reduced brittleness in comparison to ordinary concrete. Also, higher flexural strength is achieved [13,14] together with abrasion resistance, mechanical impact resistance, and resistance to freezing and thawing processes. In the production of FRC, various types of fibers are applied, differing in the material used (e.g., steel, polypropylene, carbon, glass, basalt, and natural) and the shape (e.g., straight, hooked-end, or crimped) [15,16,17,18]. Advantageous properties of FRC allow for using this material in tunnel construction, bridge surfacing, industrial flooring, heavy-duty pavements, or mining [14,19,20,21,22,23].

Also, WTSF can effectively increase concrete’s ductility, tensile strength, and compressive strength [24]; flexural behavior is also improved [25]. Zia et al. reported a 16% increase in splitting tensile strength after adding 0.3% of WTSF [26]; in the same study, an increase in water absorption up to 22% was reported after adding 0.5% of WTSF.

Many buildings and bridge structures are subjected to significant torsional moment and some of them may require strengthening. Examples of concrete elements often loaded with a torsional moment are lateral beams of stairways, edge floor joists, spatial frames, spandrel beams in buildings, beams supporting a canopy, spiral stairs, bridge decks, and reinforced concrete arches loaded perpendicularly to their surface [27,28,29,30].

Structural concrete elements submitted to a torsional moment should be properly designed and manufactured. The difference between poor-quality and good-quality concrete rests not so much on the choice of ingredients but mainly on the proportions [31]; therefore, a properly designed mixture of concrete is a key to success. This paper is devoted to studying experimentally the effect of adding two types of fiber, factory made steel fiber, and steel fiber derived from waste tires, on the torsional behavior of structural beams. Based on the literature review conducted, it appears that no analysis has been conducted on concrete elements with the addition of steel cords subjected to torsional moment. Therefore, the results of the research presented in this article are the first attempt to use waste steel fibers in elements made of cement composites subjected to twisting. These tests are the beginning of the analysis of concrete elements with steel cords subjected to torsion.

## 2. Materials and Methods

**Figure 1:**
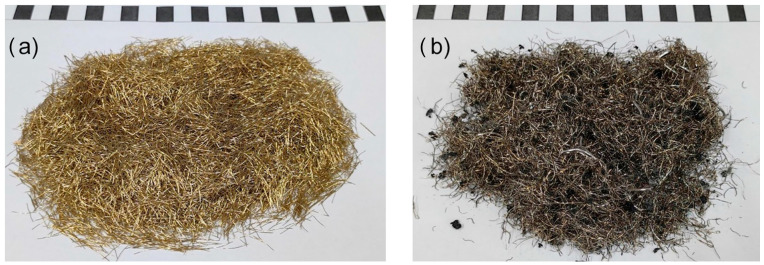
Steel fibers used in the experiment: (**a**) factory made steel fiber and (**b**) WTSF.

Three types of composite mixes were used in the experiment: control mix without any fibers (CM), fiber-reinforced composite mix containing commercial steel fibers of straight ends (steel fiber-reinforced concrete, SFRC), and fiber-reinforced composite mix containing waste steel fibers recovered from worn out tires (waste tire steel fiber-reinforced concrete, WTSFRC, see Figure 1). Table 1 presents the average diameters, lengths, and tensile strengths of commercial and waste steel fibers. The values for commercial fibers were adopted based on the manufacturer’s declaration but the values for waste fibers were tested. The measurements were carried out on 100 randomly selected waste fibers. The diameter measurements were carried out using a micrometer with a range of 50 mm and an accuracy of 0.01 mm. The length measurements were performed using a caliper with a range of 150 mm and an accuracy of 0.02 mm on previously straightened fibers. The tensile strength measurement of the fibers was conducted on a strength testing machine with a range of 10 kN and an accuracy of 1%. The standard deviation was 0.02 mm, 3.8 mm, and 3.5 MPa, respectively, for diameter, length, and tensile strength. Other components of the composite mixes were as follows: ordinary Portland cement CEM I 42.5 [32], tap water that complied with the European standard [33], and fine aggregate up to 2 mm (see Table 2).

Before preparing the concrete mixes, basic properties of the aggregate were examined. The following tests were performed: sieve analysis, fineness modulus, median diameter, loose and compacted bulk density, and water absorptivity. Sieve analysis was conducted according to EN 933-2:1996 [34]. Rectangular mesh sieves of the following mesh sizes were used: 0.63, 0.125, 0.25, 0.5, 1.0, and 2.0 mm.

Fineness modulus of the aggregate was calculated with the use of Abrams method as follows [35]:

(1) $$m_{A}=0.01\sum_{i=1}^{n}b_{i},$$

(2) $$m_{A}=n-0.01\sum_{i=1}^{n}y_{i},$$

where *n* is equal to number of nonzero *i* values, *b_i_* = 100 − *y_i_* = total percentage of the fraction of the aggregate retained on the *i*th member of the specified Tyler series of sieves, *y_i_* = total percentage of a sample of the aggregate passing through the *i*th member of Tyler series of sieves.

Median diameter is defined as follows: splitting a sample of aggregate, in such a way that half of the grains are characterized by higher or equal (to median grain) value of the diameter and the other half of grains are characterized by the lower or equal (to median grain) value of the diameter, was derived from the following formula [36]:

(3) $$d_{m}=d_{j}+\frac{d_{k}-d_{j}}{\sum f_{k}-\sum f_{j}}(50-\sum f_{i}),$$

where *d_m_* is the median grain diameter in (mm), *d_j_* is the sieve opening for Σ*f_j_*, *d_k_* is the sieve opining for Σ*f_k_*, Σ*f_j_* is the sum of the content of fractions closest to, but smaller than 50%, and Σ*f_k_* is the sum of the content of fractions closest to, but bigger than 50%.

The bulk density test of the aggregate was performed according to the EN 1097-3 standard [37]; the water absorptivity test was performed according to the EN 1097-6 standard [38].

The next stage of the research was testing the mechanical properties of the composite in a hardened state. Flexural strength was determined with the use of specimens 40 × 40 × 160 mm according to the European standard EN-196-1 [39]. During the flexural strength test, deflection was also recorded. Compressive tests were performed with the use of specimens characterized by a cross-section of 40 × 40 mm according to the standard EN-196-1 [39]. A total of 12 specimens were tested for each type of cement composite and cross-section shape. A total number of 72 specimens were tested in order to determine the compressive strength.

**Figure 2:**
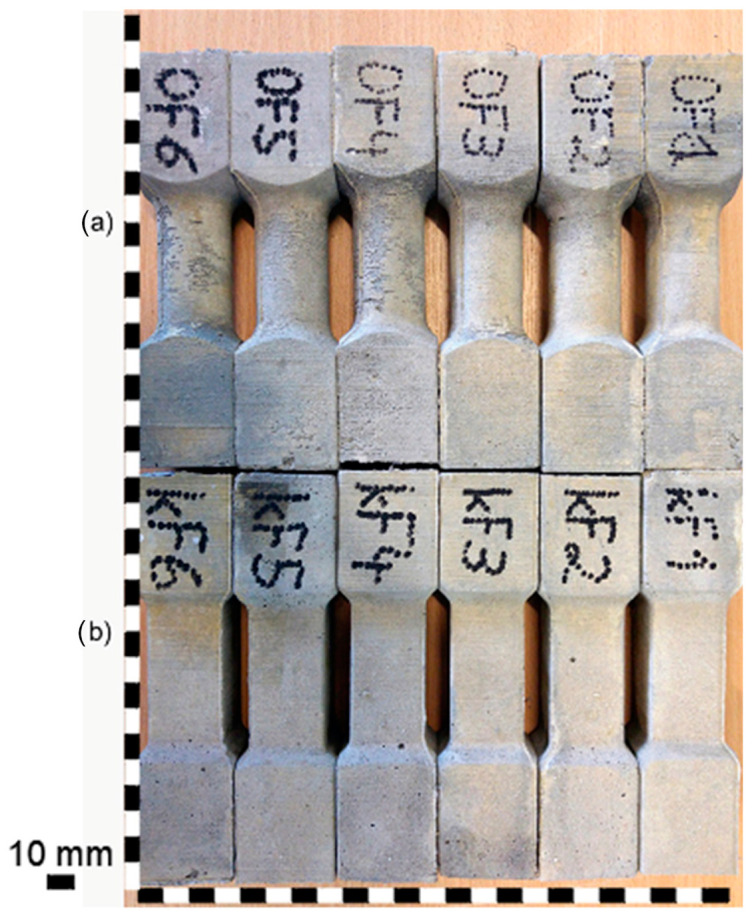
Specimens prepared for the torsion test: (**a**) circular cross-section and (**b**) rectangular cross-section.

**Figure 3:**
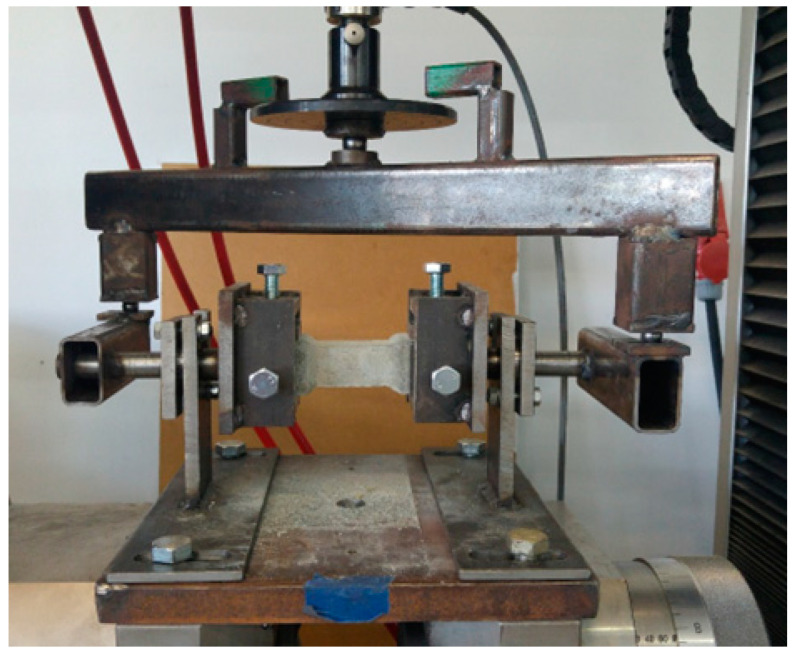
Dedicated stand for torsion test with mounted specimen.

The molds for the torsion test specimens were made from PET-G using a 3D printer. The molds consisted of two parts screwed together with 4 screws. Three samples were made from a single mold. Specimens intended for the torsion tests were characterized by a rectangular and circular cross-section and formed in such a way that the failure occurred in the narrowed zone (see Figure 2). The cross-section area of the narrowed zone was on average 716 mm^2^ and 947 mm^2^ for the circular and the rectangular cross-section shape, respectively. The specimens were mounted in the apparatus dedicated for the torsion test (see Figure 3). Six specimens were tested for each type of cement composite and cross-section shape. 72 specimens were tested in total in the torsion tests. All samples were cured in the same temperature (20 °C) and humidity (100%) conditions for 28 days. After one day, the samples were demolded.

**Figure 4:**
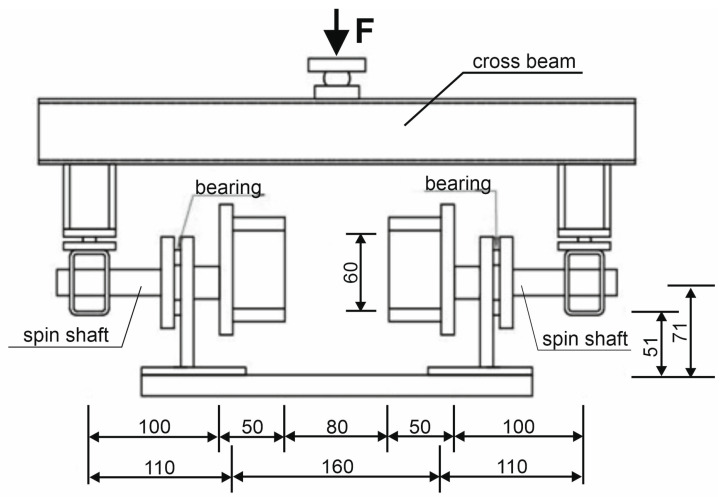
A scheme of the stand for performing torsion tests. Side view (mm).

**Figure 5:**
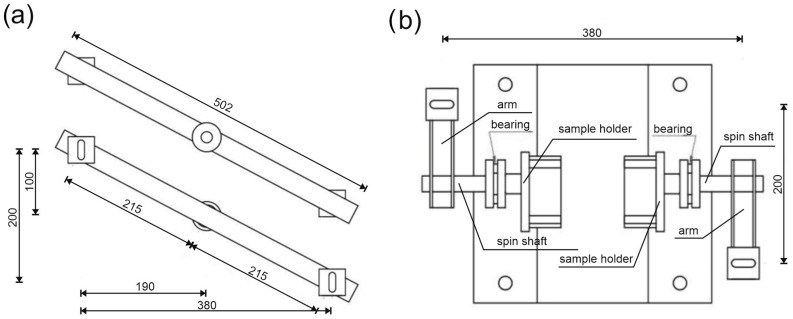
A scheme of the stand for performing torsion tests: (**a**) top and bottom view of the loading crossbeam and (**b**) top view without the loading crossbeam (mm).

The stand was composed of elements connected with articulated joints. The force was transferred from the compression testing machine to the crossbeam via a ball joint (see Figure 4). Next, the crossbeam transmitted the force into two arms via ball joints. The arms were connected to the spin shafts equipped with sample holders (see Figure 5). The testing machine with a range of 20 kN and an accuracy of 0.5% was used in the tests. The testing procedure consisted of controlling the relation between distance and speed of the machine’s traverse. Up to a displacement of 0.1 mm, the speed was 0.1 mm/min; above 0.1 mm, the speed was increased to 0.2 mm/min.

## 3. Research Test Results

**Figure 6:**
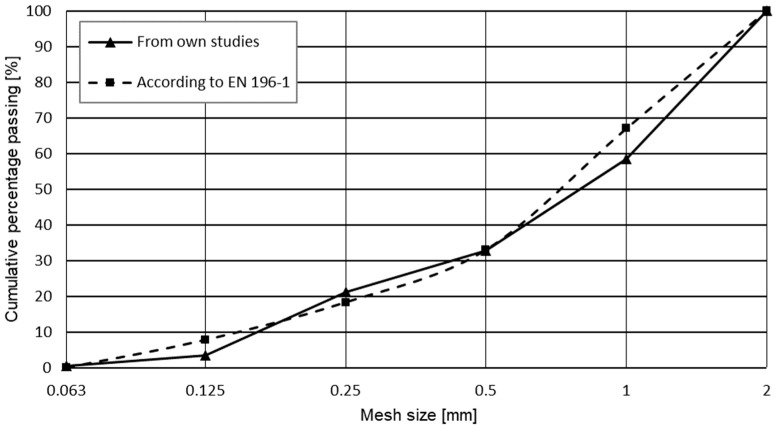
Grading curve of the aggregate used in the experiment.

The course of the grading curve of the tested aggregate (see Figure 6) deviates only slightly from the curve recommended by the European standard EN 196-1 [39].

Fineness modulus, median diameter, and bulk density in a loose and compacted state (see Table 3) are typical for the post-glacial sands found in the Western Pomerania region [36].

**Figure 7:**
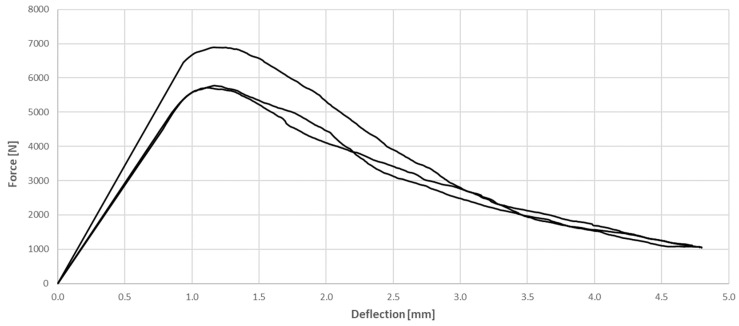
Relationship between force and deflection of the tested SFRC in flexural test.

**Figure 8:**
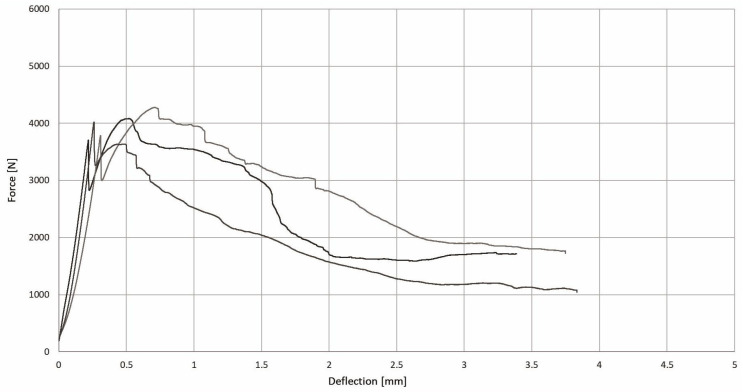
Relationship between force and deflection of the tested WTSFRC in flexural test.

Figure 7 and Figure 8 present the relationship between force and deflection of the tested SFRC and WTSFRC, respectively. The maximum force of tested specimens was achieved for SFRC; also, the curves are smoother compared to the WTSFRC results. The force–deflection relationship for the cement matrix has not been studied. The maximum values of flexural strength for all composites are provided in Table 4.

Research results of the tested composites (see Table 4) revealed that there are significant differences between the compressive strength and flexural strength of the tested composites. The composite characterized by the highest compressive strength and flexural strength was SFRC. The composite CM (without fibers) was characterized by the lowest compressive strength and flexural strength.

**Figure 9:**
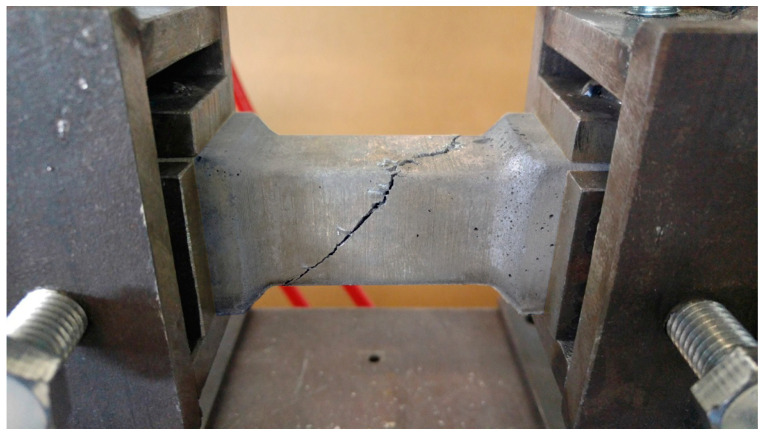
Destroyed specimen mounted in the apparatus stand after the torsion test.

**Figure 10:**
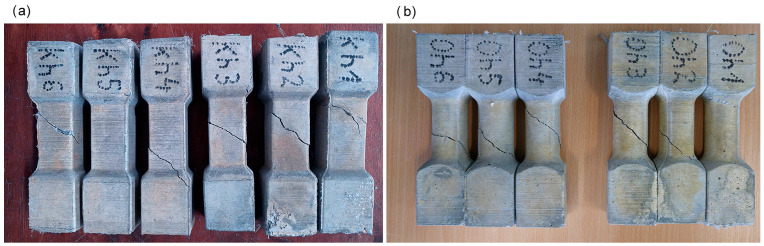
Specimens after the torsion test: (**a**) rectangular cross-section and (**b**) circular cross-section.

The destruction model of the tested specimens was similar for most of the tested specimens. After reaching the maximum value of the destructive force, a crack appeared in the sample at angle of about 45 degrees to the longitudinal axis of the specimen (see Figure 9 and Figure 10).

## 4. Discussion

**Figure 11:**
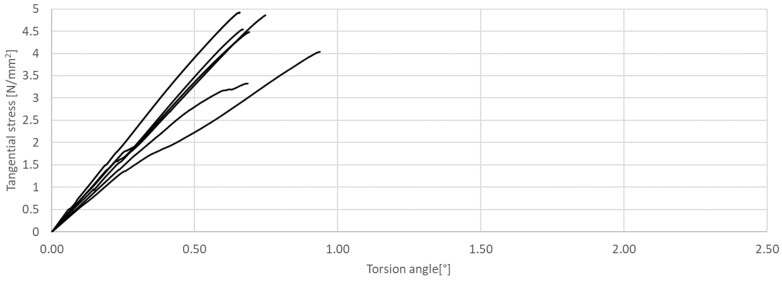
Tangential stress in correlation with torsion angle of the tested CM, rectangular cross-section.

**Figure 12:**
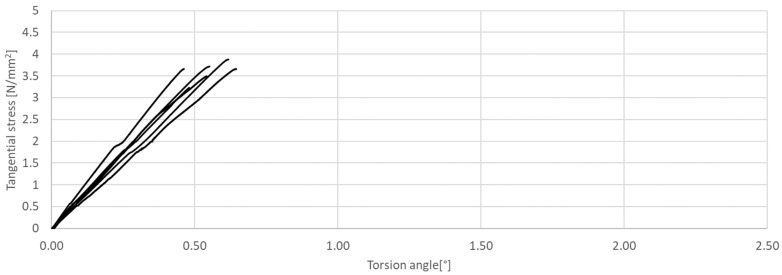
Tangential stress in correlation with torsion angle of the tested CM, circular cross-section.

**Figure 13:**
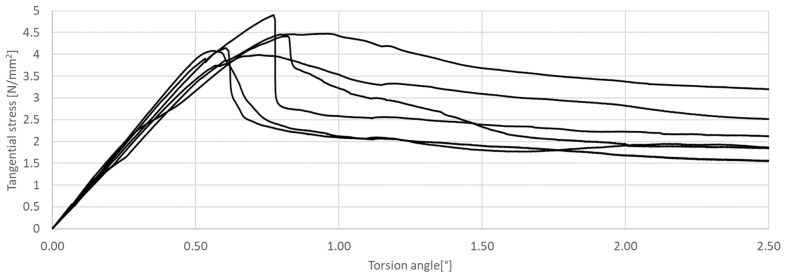
Tangential stress in correlation with torsion angle of the tested SFRC, rectangular cross-section.

**Figure 14:**
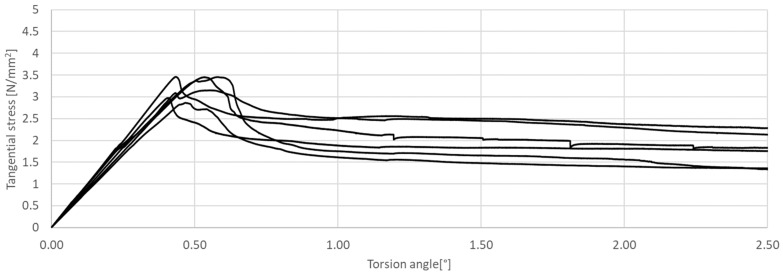
Tangential stress in correlation with torsion angle of the tested SFRC, circular cross-section.

**Figure 15:**
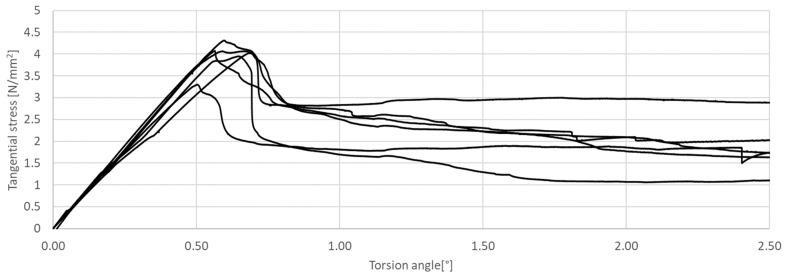
Tangential stress in correlation with torsion angle of the tested WTSFRC rectangular cross-section.

**Figure 16:**
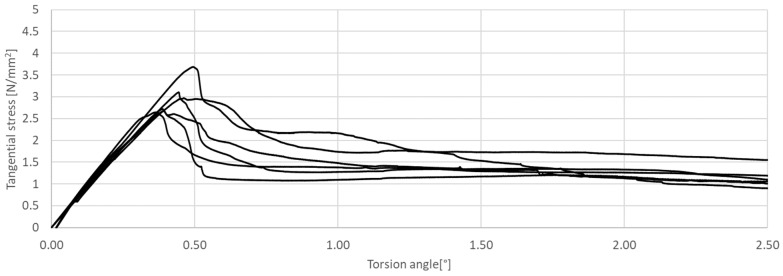
Tangential stress in correlation with torsion angle of the tested WTSFRC circular cross-section.

The research presented in this article was aimed at determining the influence of the shape of the sample’s cross-section during the torsion test. The results of the tests are presented in Figure 11, Figure 12, Figure 13, Figure 14, Figure 15 and Figure 16. The analysis of the graphs shows a few properties of the tested elements. All graphs in the initial part have a linear characteristic. This means that the torsion angle increases proportionally with the load. This is due to the fact that the elements work in the non-cracked phase. This phase ends when the linear function ends or changes its shape, for elements without and with fibers, respectively. For the samples made without fibers (Figure 11 and Figure 12), the end of the non-cracked phase is the moment when the element is destroyed. However, for elements with fibers, it is the moment (cracking moment) of transition to the next phase of its work (Figure 13, Figure 14, Figure 15 and Figure 16). Table 5 presents a comparison of cracking moments (and breaking moments—for the matrix) for all analyzed elements. The highest values of cracking/destruction moments for rectangular and circular cross-section shapes (MR and MC) were obtained for elements without fibers (CM), and the lowest values were obtained for the mixture containing waste fibers (WTSFRC). It seems that the addition of fibers to the cement matrix does not have a positive effect on the cracking moment. The positive effect of adding fibers to the cement matrix becomes visible after cracking the elements.

The shape of the sample, as well as its cross-sectional area, has an influence on the value of the cracking moment. The ratio of rectangular to circular cross-sectional area was approximately 1.32 and was similar to the 1.36 ratio of cracking moments for the cement matrix (Table 5), as opposed to composites with the addition of fibers, for which the ratio of cracking moments was about 1.5.

In Table 6, an analysis of theoretical cracking moments was conducted. Four calculation methods were applied. The ACI 318-19 [40], Hassan et al. [41], and Hsu and Mo [42] methods are based on the compressive strength of the composite, while the Okay and Engin [43] method is based on the tensile strength in bending. Depending on the calculation method used, the values of the moments differ quite significantly. The highest values were obtained for the Okay and Engin [43] method, while the lowest were for the calculation proposal contained in ACI 318-19 [40].

**Figure 17:**
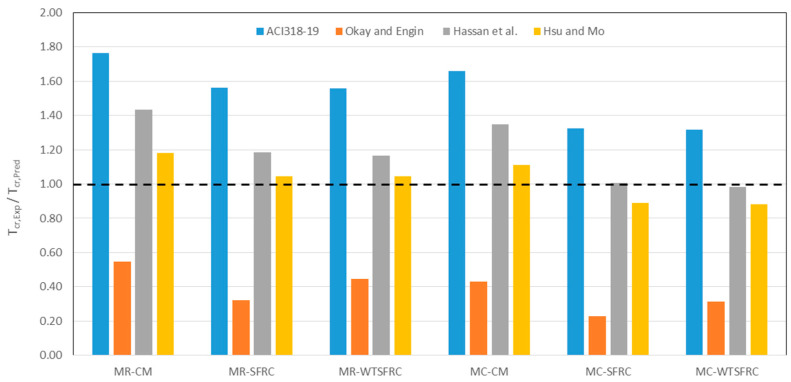
Comparison of experimental and theoretical cracking moment [40,41,42,43].

A comparative analysis of experimental and empirical cracking moments is presented in Figure 17. It shows that the moment values calculated using the ACI 318-19 [40] method are significantly lower than the experimental values obtained. Therefore, this method is not recommended for use with the composites analyzed. The calculation method proposed by Okay and Engin [43] is also not recommended for assessing the bending moments of the composites analyzed, as the theoretical values obtained are significantly higher than the experimental values. At the same time, it should be noted that the application of this method provides a safety margin. Among the other two methods, [41] and [42], the best agreement between theoretical and experimental values of cracking moments was obtained with the Hsu and Mo [42] method. This is likely due to the fact that this calculation method for cracking moments takes into account the addition of dispersed reinforcement.

Based on the cracking moments, it is possible to determine the values of tangential stress. The values of tangential stress at the moment of cracking the elements are presented in Table 7 (column *M_CR_*). In addition, the same table shows the values of tangential stresses for the torsion angle of 2.5°.

The highest values of tangential stress at the moment of cracking (TR and TC) were obtained for elements without fibers (CM), but the lowest values were obtained for the mixture containing waste fibers (WTSFRC). It seems that the addition of fibers to the cement matrix does not have a positive effect on the tangential stress. The positive effect of adding fibers to the cement matrix becomes visible after the cracking moment. For the mixtures SFRC and WTSFRC, it is possible to determine the tangential stresses. For example, for the torsion angle of 2.5°, the maximum value of the tangential stresses is obtained for elements with SFRC.

The shape of the sample has an influence on the value of the tangential stresses too. The ratio of rectangular to circular tangential stresses was between 1.20 and 1.64 (see Table 7). The lowest values of the ratio (TR/TC) were obtained for elements without fibers (CM), but the highest values were obtained for the mixture containing waste fibers (WTSFRC). The ratio of rectangular to circular areas was 1.27.

## 5. Conclusions

The conducted research tests allow for the following conclusions:

- The addition of steel fibers has little effect on compressive strength of the tested cement composites;
- The type of mixture has little effect on the cracking moment;
- Fibers significantly affect the destruction process of the cement composite, turning it from a brittle material to quasi-plastic one;
- The shape of the sample has an influence on the value of the cracking moment and the tangential stress;
- The torsional failure of the composite with the addition of steel fibers is slower in comparison to the composite without fibers. Torsional stress is partially transferred through the fibers.

## Figures and Tables

**Table 1:** Steel fibers used in the experiment.

Fiber Type	Diameter (mm)	Length (mm)	Tensile Strength (MPa)
Factory made steel fiber	0.4 ^1^	14.0 ^1^	2800 ^1^
WTSF	0.3 ^2^	13.3 ^2^	1789 ^2^

^1^ Values declared by a producer, ^2^ Mean value on the basis of measurements.

**Table 2:** Composition of the composite mixtures used in the experiment (g).

Component	CM	SFRC	WTSFRC
Cement	450	450	450
Fine aggregate	1350	1350	1350
Water	225	225	225
Steel fiber	-	60	60

**Table 3:** Properties of the aggregate used in the experiment.

Fineness Modulus via	Median	Bulk Density		Water
Abrams	Diameter	Loose	Compacted	Absorptivity
(-)	(mm)	(g/cm^3^)	(g/cm^3^)	(%)
2.77	0.84	1.70	1.84	9.2

**Table 4:** Compressive and flexural strength of the tested cement composites.

Composite Name	Compressive Strength (MPa)	Flexural Strength (MPa)
CM	43.4 (1.1) ^1^	8.5 (0.3) ^1^
SFRC	55.2 (0.8) ^1^	14.4 (1.6) ^1^
WTSFRC	48.2 (2.0) ^1^	9.7 (0.3) ^1^

^1^ standard deviation (in brackets).

**Table 5:** Average values of cracking/destruction moments (MR, MC) for the tested specimens.

Specimen Cross-Section Shape, Moment	CM	SFRC	WTSFRC
Rectangular, MR (N·m)	26.17 (2.8) ^1^	26.08 (1.9) ^1^	24.35 (2.2) ^1^
Circular, MC (N·m)	19.31 (1.4) ^1^	17.40 (1.5) ^1^	16.13 (2.4) ^1^
Aspect ratio MR/MC	1.36	1.50	1.51

^1^ standard deviation (in brackets).

**Table 6:** Cracking/destruction moments for the rectangular (MR) and circular (MC) specimens.

Calculation Methods	Equations	CM		SFRC		WTSFRC	
		MR	MC	MR	MC	MR	MC
ACI 318-19 [40]	$T_{cr}=0.33\lambda \sqrt{f_{c}}\cdot \frac{A_{cp}^{2}}{p_{cp}}$	14.82	11.64	16.72	13.12	15.62	12.26
Okay and Engin [43]	$T_{cr}=W_{t}\;f_{ct,j\;}$	47.74	45.04	80.87	76.30	54.48	51.40
Hassan et al. [41]	$T_{cr}=0.41\sqrt{f_{c}}\frac{A_{cp}^{2}}{p_{cp}}\;f_{cr}$	18.23	14.31	22.00	17.27	20.92	16.42
Hsu and Mo [42]	$T_{cr}={0.4\;A}_{cp}\;t\;\sqrt{f_{c}}$	22.15	17.38	24.97	19.61	23.34	18.32

Note: λ—modified factor (for normal-weight concrete it is equal to one); *f_c_*—compressive strength; *A_cp_*—area of the rectangular cross-section; *p_cp_*—perimeter of the rectangular cross-section; *W_t_*—section torsional resistance moment (*W_t_ = C*_1_*·h·b*^2^ for the rectangular specimens or *W_t_ = π·r*^3^/2 for the circular specimens); *b*—shorter dimension of the rectangular cross-section; *h*—longer dimension of the rectangular cross-section; *C*_1_—coefficient depending on h/b ratio (is equal to 0.208); *r*—radius of the circular specimens, *f_ctf_*—flexural strength, *f_cr_*—fiber modified factor (*f_cr_ = 1 + V_f_·A_r_/500)*; *V_f_*—percentage of the fiber dosage; *A_r_*—aspect ratio of the fiber (fiber length/fiber diameter); t—should be taken as 1.2*A_cp_/p_cp_* for solid sections.

**Table 7:** Average values of the tangential stress.

Moment or Torsion Angle	CM		SFRC		WTSFRC	
	*M_CR_*	2.5°	*M_CR_*	2.5°	*M_CR_*	2.5°
Rectangular cross-section TR (MPa)	4.36 (0.60) ^1^	- -	4.34 (0.34) ^1^	2.18 (0.59) ^1^	3.96 (0.34) ^1^	1.85 (0.59) ^1^
Circular cross-section TC (MPa)	3.62 (0.23) ^1^	- -	3.23 (0.27) ^1^	1.78 (0.39) ^1^	2.93 (0.44) ^1^	1.13 (0.23) ^1^
Aspect ratio TR/TC	1.20	-	1.34	1.22	1.35	1.64

^1^ standard deviation (in brackets).

## Data Availability

Data are contained within the article.
